# Frailty and solid-organ transplant candidates: a scoping review

**DOI:** 10.1186/s12877-022-03485-7

**Published:** 2022-11-16

**Authors:** Jonathan Kao, Natasha Reid, Ruth E Hubbard, Ryan Homes, Leila Shafiee Hanjani, Ella Pearson, Benignus Logan, Shannon King, Sarah Fox, Emily H Gordon

**Affiliations:** 1 Princess Alexandra Hospital, Metro South Hospital and Health Service, Queensland, Australia; 2 Centre for Health Services Research, The University of Queensland, Queensland, Australia; 3 School of Biomedical Science, The University of Queensland, Queensland, Australia; 4 North Metropolitan Health Service, WA Health, Western Australia, Australia; 5 The Prince Charles Hospital, Metro North Hospital and Health Service, Queensland, Australia; 6 Geriatrics and Rehabilitation Unit, Building 7 Princess Alexandra Hospital, 199 Ipswich Road, Woolloongabba, QLD 4102 Australia

**Keywords:** Frail, Frailty, Solid-organ transplant, Adverse outcome

## Abstract

**Background:**

There is currently no consensus as to a standardized tool for frailty measurement in any patient population. In the solid-organ transplantation population, routinely identifying and quantifying frailty in potential transplant candidates would support patients and the multidisciplinary team to make well-informed, individualized, management decisions. The aim of this scoping review was to synthesise the literature regarding frailty measurement in solid-organ transplant (SOT) candidates.

**Methods:**

A search of four databases (Cochrane, Pubmed, EMBASE and CINAHL) yielded 3124 studies. 101 studies (including heart, kidney, liver, and lung transplant candidate populations) met the inclusion criteria.

**Results:**

We found that studies used a wide range of frailty tools (N = 22), including four ‘established’ frailty tools. The most commonly used tools were the Fried Frailty Phenotype and the Liver Frailty Index. Frailty prevalence estimates for this middle-aged, predominantly male, population varied between 2.7% and 100%. In the SOT candidate population, frailty was found to be associated with a range of adverse outcomes, with most evidence for increased mortality (including post-transplant and wait-list mortality), post-operative complications and prolonged hospitalisation. There is currently insufficient data to compare the predictive validity of frailty tools in the SOT population.

**Conclusion:**

Overall, there is great variability in the approach to frailty measurement in this population. Preferably, a validated frailty measurement tool would be incorporated into SOT eligibility assessments internationally with a view to facilitating comparisons between patient sub-groups and national and international transplant services with the ultimate goal of improved patient care.

## Introduction

Solid-organ transplantation (SOT) has evolved from an experimental to definitive treatment for patients with end-stage liver, kidney, pancreas, heart and lung dysfunction^1^. Advances in surgical techniques and immunosuppressive therapy have seen reduced perioperative and medical complications and improved graft survival [1]. In turn, patient survival rates are high; for example, in the United States, as of 2019, one-year patient survival rates were equal to or greater than 90% for all single SOT except intestine transplantation [2]. Improvements in other patient-important outcomes, such as quality of life and functional performance, have also been reported [3]. Consequently, demand for SOT now exceeds donor organ supply and the field must explore strategies to address this balance. One such strategy is to optimize organ allocation processes through greater understanding of potential transplant candidates’ risk profiles [4, 5].

Determining transplant eligibility is a complex process undertaken by a multidisciplinary team, including transplant physicians and surgeons, specialist nurses and allied health professionals. Even though there are established scoring methods (such as the Model for End-Stage Liver Disease) and listing criteria for each organ, the decision to place a patient on the transplant waiting list often comes down to expert clinical judgement [6]. The multidisciplinary team must weigh the inherent risks of surgery and immunosuppression against the potential benefits for each individual, which in turn must be weighed against the need to ensure that there is maximum benefit derived from a finite resource. Moreover, transplant teams are increasingly being asked to evaluate older patients with more complex medical and functional needs [7]. Differences in health status, which corresponds with differences in risk of adverse outcomes, can be captured by measuring frailty [8]. Routinely identifying and quantifying frailty in potential transplant candidates would support patients and the multidisciplinary team to make well-informed, individualized, management decisions, a viewpoint shared by more than 250 kidney, liver, heart and lung transplantation specialists at the 2018 consensus conference on frailty [9].

Frailty is a state of increased vulnerability to stressors that is associated with adverse health outcomes, including death, disability and hospitalization [10, 11]. A frail individual has reduced physiological reserve and a reduced ability to compensate for disruptions to homeostasis [10]. Frailty increases with, but is not synonymous with, chronological age [12]. Frailty is prevalent in adults with organ failure and tends to develop at a younger age than the general population [13–17]. For example, in one study of dialysis-dependent patients, 73% of the entire cohort and 64% of those younger than 40 years of age were frail [18]. Even though kidney transplant candidates are likely to be a younger and healthier subset of patients with end-stage kidney disease, approximately 15% of wait-listed patients have been assessed as frail in recent studies [19, 20]. Similarly, frailty prevalence has been found to be high among younger heart failure patients, and the lack of relationship between age and frailty in this group indicates frailty in heart failure is not confined to older adults [16, 17].

Currently, there is no consensus as to a standardized tool for frailty measurement in any patient population. In a 2018 scoping review, 89 different measures were being utilized in the acute care setting alone [21]. To our knowledge, no systematic reviews have been conducted with a focus on the SOT population. The consensus conference concluded that a single frailty measure across all SOT candidates may not be appropriate due to different aspects of frailty being relevant to different patient (organ) groups [22]. However, we argue that frailty assesses intrinsic vulnerability rather than the impact of individual diseases and, as a result, there is merit in comparing frailty measurement across groups. The aim of our scoping review, therefore, was to synthesise the literature around frailty measurement in SOT candidate populations.

## Materials and methods

### Protocol and registration

This scoping review is reported according to the Preferred Reporting Items for Systematic Reviews and Meta-Analyses extension for Scoping Reviews (PRISMA-ScR) criteria [23]. The protocol was registered with Open science Framework (OSF; Digital Object Identifier 10.17605/OSF.IO/GZN38).

### Search strategy

The search strategy was developed by JK, EG and NR, with the assistance of a librarian, and conducted by JK. We searched Cochrane (Cochranelibrary.com), Pubmed (PubMed.gov), EMBASE (Embase.com) and CINAHL (EBSCOhost) databases. Search terms used were broad and included ‘transplant*’ OR ‘allog*’ (title/abstract) and ‘frail*’ (title/abstract), which we deemed would capture all types of transplants and allograft studies, as well as any studies where frailty was an intended measure, regardless of which specific measure was used. The search included all studies up until 31st July 2022, and there were no limits placed. References lists of included full-text articles were searched for additional relevant studies.

### Study selection

Studies were eligible for inclusion if they purported to measure ‘frailty’ in solid-organ (including kidney, liver, pancreas, heart or lung) transplant candidates. Additionally, studies of transplant recipients that measured frailty at, or just prior to, admission for transplant surgery were included as participants were ‘transplant candidates’ at the time of frailty assessment. Included studies could be of any design, but were to be conducted in an adult human population and published in English. Studies were excluded if they were not an original study (e.g., a protocol or review paper), were an abstract only or were not published in English. Abstract only studies were excluded as it was felt there would not be sufficient information for data analysis. Studies of non-solid organ transplant candidates (such as bone marrow transplant candidates) and studies that only measured frailty in transplant recipients were also excluded. As this review was interested in the methods by which these studies measured frailty, the measurement tool used was neither an inclusion nor exclusion criteria.

After removal of duplicates, reviewers JK and SK screened titles, abstracts and full-text of the studies. Any ambiguity regarding the study eligibility was resolved by an independent review of the study by a second and/or third reviewer (EG and/or NR).

### Data extraction and analysis

A data extraction template was devised by three reviewers (JK, NR, EG) and imported into Covidence [24]. The following data was extracted from each included article: year and country of publication, transplant organ, study design, sample size, participant sex and age, frailty measurement tool used, timing of frailty measurement, reason for measuring frailty and, where relevant, adverse outcome measures examined in relation to frailty. Frailty tools were identified as ‘established’ if they were tools specifically developed and validated as a measure of frailty in a general population. ‘Other frailty tools’ referred to all other measures, including operational definitions developed for a SOT or disease-specific group (e.g., the Liver Frailty Index) and scales not specifically developed to measure frailty (e.g., the Short Physical Performance Battery).

These are definitions that have been employed in other studies of frailty measurement [21]. No specific adverse outcomes were of interest, we sought to identify all adverse outcomes that the included studies reported on. Data was extracted for all studies, and each reviewer (NR, EG, LS, EP, BL, SK, RH, SF) was given eight to ten unique studies to extract. Another reviewer (JK) independently extracted and cross-checked data for all studies. Disagreements were resolved by consensus.

## Results

### Search and study selection

**Fig. 1:**
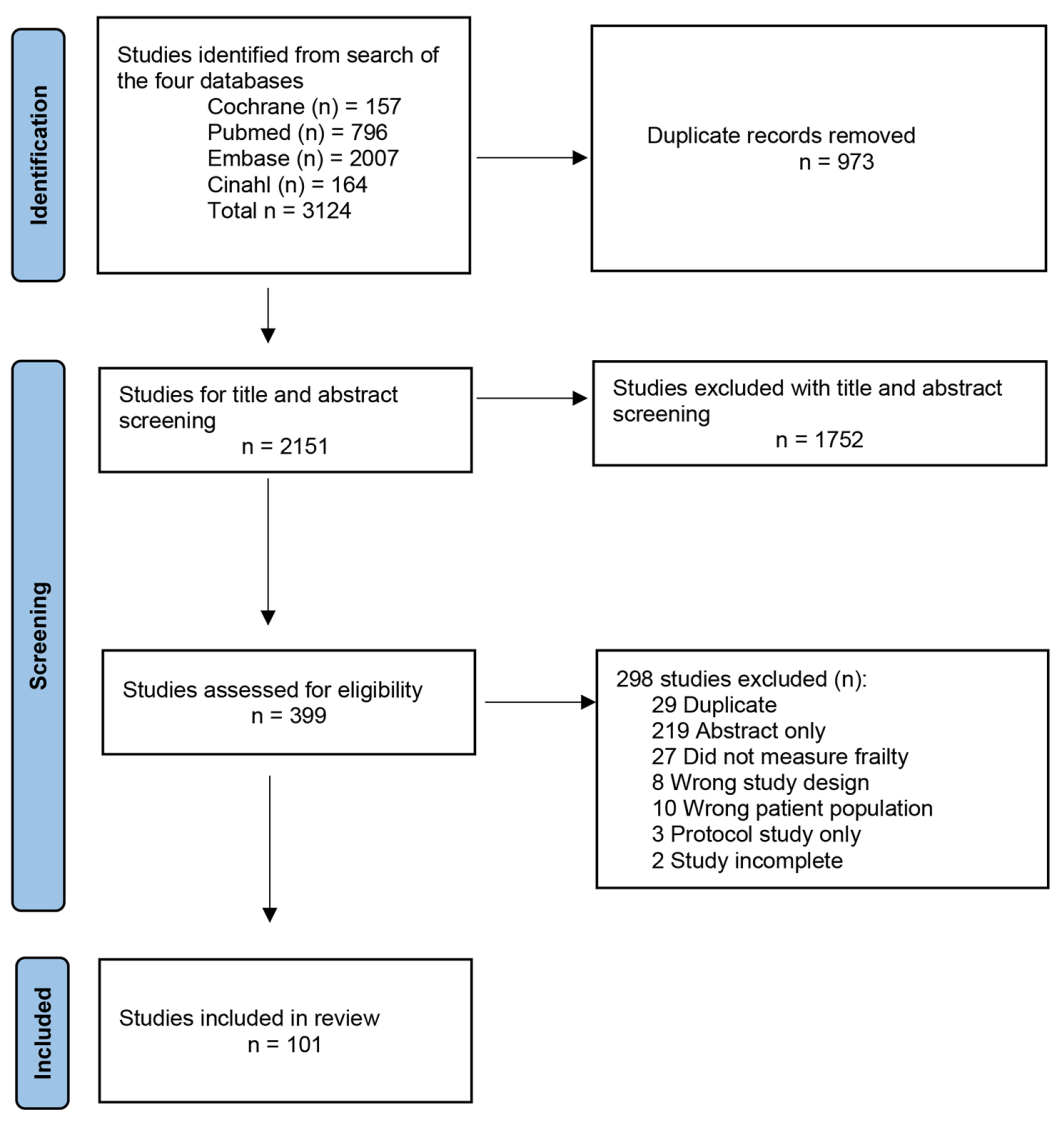
PRISMA diagram of study selection

The search strategy yielded 3124 articles, which were imported into Covidence (Fig. 1). After removal of duplicates, title and abstract screening and full-text review, a total of 101 studies were eligible for inclusion.

### Study characteristics

The characteristics of included studies are presented in Table 1. The majority of studies were conducted in kidney (36 studies; [14, 19, 20, 25–57]) and liver (36 studies; [15, 58–92]) transplant candidates. Nine studies [93–101] and 16 studies [102–117] were conducted in heart and lung transplant candidates, respectively. Four studies [118–121] included double organ transplant candidates (i.e., kidney and liver transplant candidates or heart). No studies that met our inclusion criteria have been published in pancreas transplant candidates. The majority of studies (N = 93) were published within the last seven years, with 62.4% (N = 63) published between 2018 and 2022. The majority of the studies were published in North America (N = 74). Nearly all studies were observational in nature, with only three being experimental. Study sample sizes ranged from 15 participants to over 120,000 participants. The median age of participants was 56.4 years (IQR 53.1–59.0) and the majority of participants were male (median percentage male = 60.0% (IQR 57.9–66.7)).

**Table 1:** Characteristics of the included studies

	All	Heart [93–101]	Kidney [14, 19, 20, 25–57]	Liver [15, 58–92]	Lung [102–117]	Multiorgan [118–121]
**Studies N (%)**	101 (100.0)	9 (8.9)	36 (35.6)	36 (35.6)	16 (15.8)	4 (4.0)
**Publication year N (%)**						
2019–2022	63 (62.4)	6 (66.7)	22 (61.1)	24 (66.7)	8 (50.0)	3 (75.0)
2016–2018	30 (29.7)	3 (33.3)	9 (25)	10 (27.8)	7 (43.8)	1 (25.0)
2013–2015	7 (6.9)	0	4 (11.1)	2 (5.6)	1 (6.25)	0
2012 or older	1 (1.0)	0	1 (2.8)	0	0	0
**Country N (%)**						
USA	74 (73.3)	2 (22.2)	26 (72.2)	34 (94.4)	11 (68.8)	1 (25.0)
Australia	9 (8.9)	5 (55.6)	0	1 (2.8)	3 (18.8)	0
Japan	1 (1.0)	0	1 (2.8)	0	0	0
Italy	2 (2.0)	1 (11.1)	0	0	0	1 (25.0)
Canada	2 (2.0)	0	1 (2.8)	0	1 (6.25)	2 (50.0)
Poland	1 (1.0)	0	0	0	1 (6.25)	0
Netherlands	2 (2.0)	0	2 (5.6)	0	0	0
Brazil	1 (1.0)	0	1 (2.8)	0	0	0
Spain	4 (4.0)	1 (11.1)	3 (8.3)	0	0	0
Hong Kong	1 (1.0)	0	1	0	0	0
France	1 (1.0)	0	1	0	0	0
Germany	1 (1.0)	0	0	1 (2.8)	0	0
**Number of participants**						
# of studies reporting	101	9	36	36	16	4
Range (N)	15–125,304	37 − 36,790	18–125,304	50 − 24,505	15–618	47 − 16,301
**Age of participants**						
# of studies reporting	98	8	34	36	16	4
Range (years)	31.0–64.0	51.0–60.0	44.7–61.8	51.0–64.0	31.0-62.9	51.2–61.0
Median (IQR)	56.4 (53.1–59.0)	53.0 (53.0-53.5)	53.9 (53.0-58.5)	58.0 (56.4–60.0)	57.9 (54.8–59.0)	54.6 (52.6-573.3)
**Percentage of males**						
# of studies reporting	97	8	34	35	16	4
Range	42.9–96.1	65.0–80.0	42.9–96.1	54.0-68.8	47.7–82.0	53.0-80.8
Median (IQR)	60.0 (57.9–66.7)	70.0 (68.0-74.5)	62.0 (60.0-63.9)	59.0 (57.6–64.5)	56.0 (51.5–58.0)	64.0 (61.0-68.2)
**Study design N (%)**						
Qualitative	0	0	0	0	0	0
Experimental	3 (3.0)	1(11.1)	1 (2.8)	0	1 (6.25)	0
Observational	98 (97.0)	8 (88.9)	35 (37.2)	36 (100.0)	15 (93.8)	4 (100.0)

### Frailty measurement

Frailty measurement characteristics of the included studies are presented in Table 2. Most studies measured frailty at time of transplant eligibility assessment (N = 27) or at admission to hospital for transplant surgery (N = 16). Ten studies measured frailty retrospectively in transplant recipients and eight studies measured frailty when the participant was added to the transplant waitlist. Fifteen studies measured frailty at other times, such as four weeks after the participant was added to transplant waitlist. There were 18 studies that did not specify the point at which frailty was measured during the participant’s transplant journey. Seven studies measured frailty at more than one time point.

**Table 2:** Frailty measurement

	All	Heart [93–101]	Kidney [14, 19, 20, 25–57]	Liver [15, 58–92]	Lung [102–117]	Multiorgan [118–121]
**Studies N (%)**	101 (100.0)	9 (8.9)	36 (35.6)	36 (35.6)	16 (15.8)	4 (4.0)
**When was frailty measured N (%)**						
At assessment for transplant eligibility	27 (26.7)	4 (44.4)	11 (30.6)	7 (19.4)	4 (25.0)	1 (25.0)
When the patient was added to the transplant wait-list	8 (7.9)	0	3 (8.3)	2 (7.2)	3 (18.8)	0
At admission to hospital for transplant surgery	16 (15.8)	1 (11.1)	14 (38.9)	1 (3.3)	0	0
Retrospectively in transplant recipients	10 (9.9)	0	1 (2.8)	6 (16.7)	0	3 (75.0)
The time-point(s) was not reported	18 (17.8)	2 (22.2)	3 (8.3)	9 (25.0)	4 (25.0)	0
Other time-point^a^	15 (14.8)	1 (11.1)	1 (2.8)	8 (22.2)	5 (31.3)	0
More than 1 time-point^b^	7 (6.4)	1 (11.1)	3 (8.3)	3 (8.3)	0	0
**Number of frailty measurement tools used per study N (%)**						
One frailty measurement tool	83 (82.2)	6 (66.6)	32 (88.9)	30 (83.3)	11 (68.8)	4 (100.0)
Two or more frailty measurement tools	18 (17.8)	3 (33.3)	4 (11.1)	6 (16.7)	5 (31.3)	0
**Total number of frailty measurement tools used N (%)**	123 (100.0)	12(9.8)	41 (33.3)	43 (35.0)	23 (18.7)	4 (3.3)
**Type of frailty measurement tools N (%)**						
Established frailty tools	63 (51.2)	8 (66.7)	32 (78.0)	9 (20.9)	13 (56.5)	1 (25.0)
Other frailty tools^c^	60 (48.8)	4 (33.3)	9 (22.0)	34 (79.1)	10 (43.5)	3 (75.0)
**Frailty measurement tool used N (%)**						
*Established frailty tools*						
Clinical Frailty Scale	3 (2.4)	0	1 (2.4)	1 (2.3)	1 (4.3)	0
Fried Frailty Phenotype (Standard and Modified)	55 (44.7)	8 (66.7)	28 (68.3)	8 (18.6)	11 (47.8)	0
Frailty Index	3 (2.4)	0	1 (2.4)	0	1 (4.3)	1 (25.0)
Groningen Frailty Indicator	2 (1.6)	0	2 (4.9)	0	0	0
*Other frailty tools*						
Liver Frailty Index	19 (15.4)	0	0	19 (44.2)	0	0
Short Physical Performance Battery	10 (8.1)	2 (16.7)	0	0	7 (30.4)	1 (25.0)
Karnofsky Performance Status	4 (3.3)	0	0	3 (7.0)	0	1 (25.0)
OHT frailty screening tool	1 (0.8)	1 (8.3)	0	0	0	0
The frailty risk score	2 (1.6)	1 (8.3)	1 (2.4)	0	0	0
Kihon checklist criteria	1 (0.8)	0	1 (2.4)	0	0	0
Functional metrics^d^	10 (8.1)	0	2 (4.9)	8 (18.6)	0	0
CES depression scale	1 (0.8)	0	1 (2.4)	0	0	0
Montreal Cognitive Assessment (MoCA)	2 (1.6)	0	1 (2.4)	0	1 (4.3)	0
Multidimensional prognosis index	1 (0.8)	0	1 (2.4)	0	0	0
FRAIL scale	2 (1.6)	0	2 (4.9)	0	0	0
Braden scale	1 (0.8)	0	0	1 (2.3)	0	0
A comprehensive frailty severity index	1 (0.8)	0	0	1 (2.3)	0	0
5 item Self-reported test	1 (0.8)	0	0	1 (2.3)	0	0
Muscle wasting	1 (0.8)	0	0	1 (2.3)	0	0
DMI-10	1 (0.8)	0	0	0	1 (4.3)	0
Combined frailty	1 (0.8)	0	0	0	1 (4.3)	0
Frailty index for people living with HIV	1 (0.8)	0	0	0	0	1 (25.0)
**Prevalence of frailty**						
# of studies reporting	82	9	29	27	15	2
Range (%)	2.7–100.0	2.7–78.4	11.2–66.2	14.0–47.0	12.0–45.0	9.0-100.0
**Purpose of measuring frailty N**						
Risk stratification^e^	74	6	25	28	11	4
Study inclusion/exclusion criteria	2	0	1	0	1	0
Transplant inclusion/exclusion criteria	2	0	2	0	0	0
Outcome measure^f^	13	1	2	5	4	1
Descriptive^g^	21	2	8	8	2	1
Feasibility^h^	8	1	3	3	1	0
Other	1	0	0	0	1	0

^a^ Frailty measured at other time-points (e.g., from 1 day prior to transplant to within 30 days after transplant listing)

^b^ Studies that measured frailty at more than 1 point (e.g. at assessment for transplant eligibility, then at admission to hospital for transplant)

^c^ Tools not designed or validated to measure frailty in general population (e.g., operational definitions developed for a SOT or disease-specific group and scales not specifically developed to measure frailty

^d^ Functional metrics – Combination of metrics that do not fit with established frailty tool (grip strength, 30 s chair sit-stand, sit -reach, timed up and go, gait speed)

^e^ Studies that examine the relationship between frailty and adverse health outcomes

^f^ Studies that measure frailty as an outcome (e.g., impact of novel drug on frailty in patients awaiting transplant)

^g^ Studies that report the prevalence/other characteristics of frailty in a sample

^h^ Studies that assess if a frailty tool can feasibly be used in their service

Overall, 22 different frailty measurement tools were used 123 times in the 101 included articles. The majority of studies used one frailty measurement tool (N = 83, 82.2%) and 18 studies utilized two or more frailty measurement tools. Of the 22 different measures used, four were ‘established frailty tools’ used in 51.2% of cases (N = 63) and the other 27 were ‘other frailty tools’ used in 48.8% of cases (N = 60). Descriptions of the frailty measurement tools are presented in Table 3. The most commonly used frailty tool across all included studies was the Fried Frailty Phenotype (standard and modified; N = 55/123, 44.7%). However, in liver transplant studies, the most commonly used frailty tool was the Liver Frailty Index (N = 19/43, 44.2%). Other established tools included the Clinical Frailty Scale, Rockwood’s Frailty Index and the Groningen Frailty Indicator. Other tools included validated scales such as the Short Physical Performance Battery and physical metrics such as grip-strength.

Eighty-two studies reported a frailty prevalence rate. Prevalence ranged from 2.7% in heart transplant candidates [96] to 100% in HIV-positive liver transplant candidates [118]. Risk stratification was the most common reason for frailty measurement (N = 74). Twenty studies had more than one purpose for frailty measurement. For example, some studies evaluated the feasibility of incorporating frailty assessment into transplant eligibility assessment as well as estimated frailty prevalence and its associated risk of adverse outcomes.

**Table 3:** Description of frailty measurement tools

‘Established’ frailty measurement tools^a^	Description
*Multidimensional* ^*b*^	
Frailty Index	Calculated by counting the number of deficits across multiple domains out of a total list of potential deficits for that person.
Groningen Frailty Indicator	Tool consisting of fifteen questions embedded within the questionnaire to assess the prevalence of frailty. This instrument is aimed at determining the level of frailty through measuring loss of function in four domains: physical (mobility functions, multiple health problems, physical fatigue, vision and hearing), cognitive (cognitive dysfunction), social (emotional isolation) and psychological (depressed mood and feelings of anxiety).
Clinical Frailty Scale	A 9-point scale that summarizes the overall level of fitness or frailty of an older adult.
*Not multidimensional* ^*c*^	
Fried Frailty Phenotype	Defines frailty as the presence of five components: weakness, slowness, exhaustion, low physical activity, and unintentional weight loss.
**Other frailty measurement tools** ^**d**^	
*Multidimensional*	
Kihon Checklist	25-item questionnaire including seven categories: daily life, physical ability, nutrition, oral condition, the extent to which one is housebound, cognitive status, and depression risk.
OHT (Orthoptic heart transplantation) Frailty Screening Tool	Objective frailty tool developed for patients undergoing orthotopic heart transplantation incorporating age, BMI, comorbidities, laboratory values and functional status.
Multidimensional prognosis index	A predictive tool of mortality for hospitalised elderly patients based on a standardised Comprehensive Geriatric Assessment. It is based on information on functional, cognitive, and nutritional status, as well as medical and social factors.
Comprehensive frailty severity index	Derived from well documented surrogates of physical performance (Karnofsky performance scale), Nutrition (Modified Academy/ASPEN assessment), and severity of liver disease and inflammation (CONUT score).
Combined frailty	Composite 7-item measure, which includes modified Fried Frailty phenotype and domains of both depression and cognitive impairment
*Not multidimensional*	
Liver Frailty Index	Tool composed of 3 performance-based tests (grip strength, chair stands, and balance).
Short Physical Performance Battery	An objective assessment tool for evaluating lower extremity functioning in older persons. Consists of 3 components measured, ability stand in different positions, timed walking trials, sit to stand times.
Karnofsky Performance Status	Assessment tool for functional impairment. A 0-100 scale that summarizes the physical function of a patient
Braden Scale	Developed for early identification of patients at risk for forming pressure sores. The scale is composed of six subscales that reflect: sensory perception, skin moisture, activity, mobility, friction and shear, and nutritional status.
The frailty risk score	Composed of 16 biopsychosocial factors including fatigue, weakness, dyspnea, chronic pain, falls, vision impairment, urinary incontinence, and nutrition issues plus biomarkers: C-reactive protein, white blood cell count, hemoglobin, and albumin.
CES (Center for Epidemiological Studies) depression scale	A 20-item measure that asks caregivers to rate how often over the past week they experienced symptoms associated with depression, such as restless sleep, poor appetite, and feeling lonely.
Montreal Cognitive Assessment (MoCA)	A cognitive screening test consisting of 30 questions. Testing the domains of Orientation, Memory, Executive function/visuospatial ability, Language, Abstraction, Animal naming, Attention and clock-drawing test.
FRAIL scale	Five self-reported questions assessing fatigue, resistance, ambulation, illness, and loss of weight
5 item Self-reported test	5 item test of physical frailty including: Unintentional weight loss, exhaustion, physical activity, activities of daily living, and instrumental ADLs
Muscle wasting	Muscle measurements collected from CT scans. Psoas muscle size (cross-sectional area, in mm2) and quality (density, Hounsfield units [HU]), which included both left and right psoas muscles, were measured at the L4 vertebral level.
DMI-10 (depression in the medically ill)	The DMI-10 is a 10-item questionnaire that is designed to measure depression in the medically ill and avoids the use of items such as fatigue, sleep, appetite disturbance and weight change that are common to both depression and many illnesses.
Frailty index for people living with HIV [137]	A frailty index designed for patients with HIV. Contains 30 relatively nonspecific health variables including co-morbidities, BMI, biochemistry, HIV viral load, CD4 + count.
Functional metrics	
Grip Strength	A simple measurement of grip strength.
Gait Speed	A simple measure of gait speed.
30-Second Chair Sit-Stand	A count of how many stands from a chair a person can complete in 30 s.
Timed Up and Go	The time it takes to stand from a sitting position, walk three metres, turn around and sit back down.

^a^ Frailty tools are considered ‘established’ if they were tools specifically developed and validated as a measure of frailty in a general population

^b^ Frailty measurement tools that assess deficits across multiple domains including cognition, function, sensorium, nutrition and co-morbidity

^c^ Frailty measurement tools that assess deficits in single domains

^d^ Frailty tools that did not meet the ‘established’ criteria above are listed as ‘other frailty measurement tools’

### Adverse outcome measures examined in relation to frailty

**Fig. 2:**
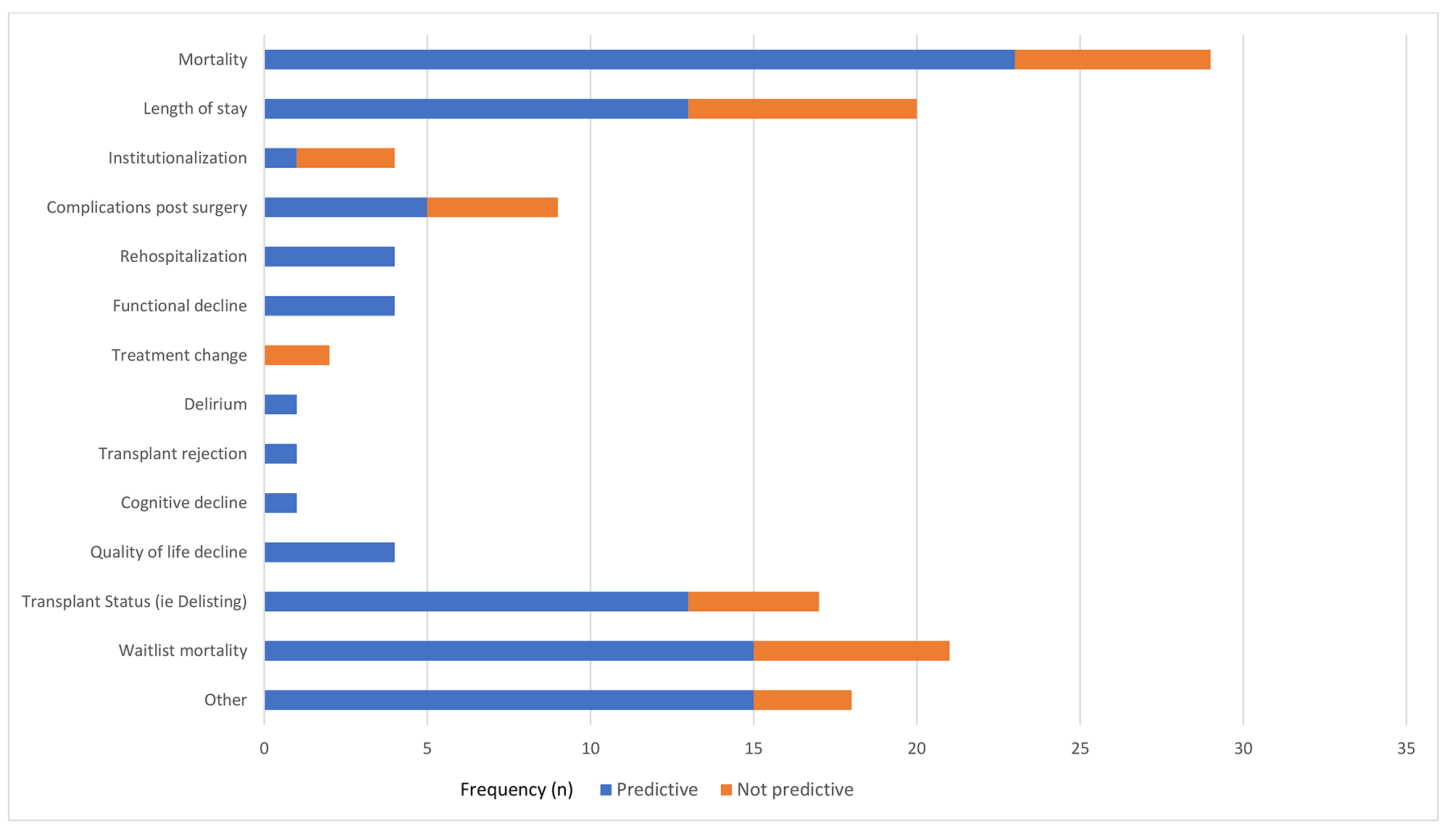
Association between frailty and adverse outcomes. (*Note: ‘Predictive’ = statistically significant association between adverse outcome and pre-transplant frailty*)

The association between frailty and adverse outcomes was investigated in 74 studies. The majority of these studies measured more than one adverse outcome, which are listed in Table 4. The most commonly measured adverse outcomes were mortality (N = 29), hospital length of stay (N = 20), waitlist mortality (N = 21) and transplant status (e.g., de-listing; N = 17). Of these 74 studies, 70 (94.6%) found that pre-transplant frailty was predictive of at least one adverse outcome, where those who were frail (or had higher levels of frailty) were more likely to experience an adverse event (Fig. 2). For example, frailty was predictive of mortality in 23 of the 29 studies (79.3%) examining this outcome.

**Table 4:** Adverse outcome measures examined in relation to frailty

	All	Heart [93–98]	Kidney [14, 19, 20, 25–49]	Liver [15, 58–86]	Lung [102–116]	Multiorgan [118–120]
**# of studies examining at least one adverse outcome N (%)**	74 (73.3)	6 (5.9)	25 (24.8)	28 (27.7)	11 (10.9)	4 (4.0)
**# of studies examining each adverse outcome N** Mortality	29	5	6	9	5	4
Length of stay^a^	20	4	4	5	5	2
Institutionalization^b^	4	0	1	2	0	1
Complications post-surgery	9	0	3	4	1	1
Rehospitalization^c^	4	0	2	1	1	0
Functional decline	4	0	1	1	1	1
Treatment change^d^	2	2	0	0	0	0
Delirium	1	0	1	0	0	0
Transplant rejection^e^	1	0	0	1	0	0
Cognitive decline	1	0	1	0	0	0
Quality of life decline	4	0	2	1	1	0
Transplant Status (i.e., Delisting)	17	3	6	5	2	1
Waitlist mortality	21	1	6	12	2	0
Other	18	1	10	4	3	0

^a^ Length of stay during transplant admission

^b^ Discharge to a care facility

^c^ Representation post-discharge after transplant

^d^ Additional procedure/treatment (e.g., Ventricular Assisted Device insertion)

^e^ Transplant rejection/acute cellular rejection

## Discussion

This scoping review synthesizes the literature around frailty measurement in solid-organ transplant candidates. We found this to be an emerging field of research, with most studies conducted in kidney and liver transplant candidates in North America in the last seven years. Most studies were observational and examined the relationship between frailty and adverse outcomes, particularly mortality and hospital length of stay. Overall, there were 22 different frailty measures used across the 101 studies, including four ‘established frailty tools’. The two most commonly used tools were the Fried Frailty Phenotype and Liver Frailty Index. Frailty prevalence estimates for this middle-aged, predominantly male, population varied widely, from 2.7 to 100%.

In this review, we aimed to include any article claiming to measure frailty to ascertain the full range of frailty measurement tools utilized in the SOT literature. We demonstrated that whilst many articles purport to measure frailty, almost half did not use an established frailty measurement tool. Using non-established measures to identify frail patients is not unique to this population, and is problematic as it constrains the generalisability of studies [122].

Defined by Fried et al. in 2001, the Fried Frailty Phenotype conceptualizes frailty as a clinical syndrome (a set of signs and symptoms that tend to occur together, thus characterizing a specific medical condition) [123]. More specifically, it identifies frailty as the presence of ≥ 3 of 5 criteria: unintentional weight loss of ≥ 10lbs in the preceding year, self-reported exhaustion, weak grip strength, slow walking speed, low physical activity [123]. It is a well-known and widely-used tool in the non-transplant frailty literature and it has many strengths: it is clinically coherent, reproducible, and identifies frailty as a wasting disorder with sarcopenia as a key pathophysiological feature [123]. The most commonly used frailty measurement tool in liver transplant candidates, the Liver Frailty Index, also focuses on physical signs and symptoms of frailty. Developed only four years ago, it comprises three performance-based tests (of grip strength, sit-to-stand transfers and balance) and was originally designed to capture extrahepatic complications of cirrhosis and to enhance mortality prediction in patients with cirrhosis [64].

While the physical manifestations of frailty are important, the omission of disorders of cognition and mood from frailty assessment is controversial: frailty in the clinical setting consists of more than weakness, slowness and wasting [124]. In the non-transplant literature, an alternative conceptualization of frailty is a multidimensional risk state which can be quantified by the number rather than by the nature of health problems [125, 126]. The Frailty Index (FI) model, defined by Rockwood and colleagues, employs a well-defined methodology to create an index as a proportion of deficits [127]. Since FIs can be constructed from different numbers and types of deficits, a measure of frailty status can be derived from information routinely collected during patient assessment and, as a result, it is often touted as the ‘ideal’ frailty measure in the clinical setting. The FI methodology allows for organ-specific deficits to be included and as a result it may be a suitable standardized measure that addresses the concern that not all frailty measures are applicable to all patient (organ) groups [9], An added advantage of using the FI in studies of SOT populations would be the ability to compare prevalence levels and adverse outcomes across groups, which would unify the literature and generate greater impact and advocacy. There are many other tools that measure the ‘multidimensionality’ of frailty. For example, the Clinical Frailty Scale (CFS), also defined by Rockwood [128], is a judgement-based tool that estimates frailty severity according to increasing morbidity and functional dependence. Despite the prominence of these tools in the frailty literature, this scoping review found that they are infrequently utilized in the SOT population.

Different frailty tools do yield different estimates of prevalence. For example, in a meta-analysis of general population-based data from 62 countries and territories, frailty prevalence estimates ranged between 12% (Fried Frailty Phenotype) and 24% (Frailty Index) in adults aged 50 years and over [129]. While there is some overlap in identification of frailty, it is likely that the phenotypic and multidimensional models (and their associated measurement tools) capture different patient groups [130]. In the SOT literature, prevalence rates vary greatly, reflecting between-study methodological differences in sample size and participant inclusion criteria (such as HIV positive status) as well as frailty measurement tools. It is possible that the predominance of phenotypic frailty tools in this field of research may result in an underestimation of frailty burden in the SOT population. This in turn may lead to an underestimation of risk in this population. Consequently, we would further advocate that future SOT studies utilize an established multidimensional frailty tool, such as Rockwood’s Frailty Index, in their assessment of frailty in potential candidates.

In the SOT candidate population, this scoping review found frailty to be associated with a range of adverse outcomes, with most evidence for increased mortality (including post-transplant and wait-list mortality), post-operative complications and prolonged hospitalisation. Fewer studies explored the relationship between frailty and patient-centered outcomes, such as functional and cognitive decline, which are of critical importance to the informed decision-making process. In the general population literature, there has been an extensive evaluation of the ability of frailty tools to predict adverse outcomes, particularly survival. In comparison studies (wherein multiple frailty measures are applied to the same patient population), predictive power varies between tools, with a trend towards multidimensional frailty tools having higher predictive power for short-term survival [131]. At the present time there is limited data to compare the predictive validity of frailty tools in the SOT population.

SOT studies also varied greatly with respect to the timing of frailty measurement relative to transplantation. We would argue that measurement of frailty at transplant eligibility assessment would be most clinically informative, yet only approximately one quarter (N = 27; 26.7%) of studies measured frailty at this time-point. In the kidney transplant population, frail candidates experience a greater deterioration in health-related quality of life and face a higher risk of mortality while awaiting a transplant when compared with non-frail candidates [25, 132]. Similar associations between frailty, reduction of quality of life and wait-list mortality have been found in heart, lung, and liver transplant candidates [60, 69, 133–135]. Overall, frail candidates are less likely to receive a transplant than non-frail candidates [136]. While frailty at time of kidney transplantation has been associated with several post-transplantation complications, including delirium, prolonged hospitalisation, delayed graft function, immunosuppression intolerance and mortality [22], it remains unclear how frailty at time of transplant eligibility assessment relates to these outcomes.

This scoping review has a number of limitations. Firstly, the scope of this review was very broad, not only in terms of organ systems, but also frailty measures and time-points. As a result, quality assessments and meta-analysis were not conducted. Further, if a study used other measures of physical function, such as muscle strength or gait speed tests, but did not claim to measure frailty itself, it would not have been identified by our search. Additionally, our search terms were conducted in title/abstract only, however we consider this strategy would have captured almost all relevant studies. Secondly, non-English studies were not included and the grey literature was not reviewed. Thirdly, initial screening was performed by mainly one rather than two reviewers.

The broad scope of this review is also a strength. By synthesizing frailty data for all SOT groups, clinicians and researchers will be able to consider the evidence relevant to their specialty. It highlights gaps in frailty research in this field, such as the lack of experimental and qualitative studies addressing frailty and the small number of studies utilizing established multidimensional frailty tools such as the Frailty Index. Furthermore, it emphasizes the need for future research to address more focused research objectives such as determining the predictive power of different frailty tools in different organ groups for standard medical/surgical as well as patient-centered outcomes.

Ultimately, identifying frailty in potential SOT candidates may improve patient care. While the primary benefit may be in risk-stratification (i.e., defining an individual’s risk profile so to inform the decisions of patients and specialists), it is possible that, in the future, frailty measurement may also shed light on potentially reversible factors amenable to targeted interventions pre- and post-transplant. At a health service level, identifying frailty in SOT candidates has the potential to influence the balance of organ supply and demand by informing the clinician and the patient regarding the risks and benefits of transplantation. Since misclassification of frailty may have significant, negative implications for the patient and the health service, relying solely on clinical acumen is not ideal. Preferably, a validated frailty measurement tool would be incorporated into SOT eligibility assessments internationally. This would facilitate comparisons between patient sub-groups and national and international transplant services and would permit large-scale synthesis of relevant data, which in turn would foster improvements in SOT patient care.

## Data Availability

All data generated or analysed during this study are included in this published article.

## Abbreviations

CFS: Clinical Frailty Scale.
CINAHL: Cumulative Index to Nursing and Allied Health Literature.
EMBASE: Excerpta Medica Database.
FI: Frailty Index.
OHT: Orthoptic Heart Transplant.
OSF: Open Science Framework.
PRISMA: Preferred Reporting Items for Systematic Reviews.
PRISMA-ScR: Preferred Reporting Items for Systematic Reviews and Meta-Analyses extension for Scoping Reviews.
SOT: Solid Organ Transplant.
